# Fear of missing out (FoMO) mediate relations between social self-efficacy and life satisfaction

**DOI:** 10.1186/s41155-021-00193-w

**Published:** 2021-08-23

**Authors:** Metin Deniz

**Affiliations:** 1 Department of Educational Sciences, Guidance and Psychological Counseling, Bartin University, Bartin, Turkey; 2 Department of Guidance and Psychological Counseling, Faculty of Education, Bartiın University, Bartin, Turkey

**Keywords:** FoMO, Social self-efficacy, Life satisfaction, Undergraduates, Turkey

## Abstract

The purpose of this study was to examine whether fear of missing out (FoMO) mediate relations between social self-efficacy and life satisfaction among undergraduates. The participants involved 323 undergraduates (female, 66.3%; male, 33.7%). The age of participants ranged between 18 and 32 years (*M* = 21.52, *SD* = 2.69). The study data was gathered using the Fear of Missing out Scale, the Social Efficacy and Social Outcome Expectation Scale and the Satisfaction with Life Scale. The research data were analyzed using the structural equation model and bootstrapping method. As a result of the structural equation model, FoMO mediates the relationship between social self-efficacy and life satisfaction. As a result of the bootstrapping analysis, it was seen that all direct and indirect effects are significant. The results, recommendations, and limitations of the study were discussed.

## Introduction

Social networks have become an important part of our lives with the developments in technology. Thanks to social communication networks, individuals can receive instant news about what others are doing. Moreover, the individual can keep others informed about what is happening at that moment in his life. The continuous accessibility of social networks provides the opportunity for a significant increase in options for connecting, sharing, and having experiences with acquaintances and friends (Fuster et al., 2017). The individual also meets the need to socialize by making shares on social networks and following the posts made. However, the individual can meet his/her socialization need in unhealthy ways. The need for socialization not met in healthy ways can prepare an environment for the individual to experience FoMO (fear of missing out). FoMO is a concept that is associated with many characteristics of the individual. In this study, the mediating role of FoMO in the relationship between social self-efficacy and satisfaction with life was examined.

## Fear of missing out

Przybylski et al. (2013) associated FoMO with the self-determination theory (Deci & Ryan, 1985), arguing that it arises from the psychological needs of the individual such as autonomy, competence, and relatedness. FoMO is defined as “a pervasive apprehension that others might be having rewarding experiences from which one is absent” (Przybylski et al., 2013, p.1841). This anxiety creates an environment for the individual to stay in touch and communicate with their social environment in order not to miss anything out (Oberst et al., 2017; Wiesner, 2017). The individual constantly desires to be informed about what others are doing now and there, for fear of missing out on developments (Przybylski et al., 2013). The more activities the person can do or situations they can experience, the less likely it is to choose the best option. This situation will cause that person to question how much of his own choice is “the best choice” (Milyavskaya et al., 2018). As a result, the person will start to feel anxiety by thinking that there may be potential activities or places other than what they are in (Przybylski et al., 2013). The fear of missing out on developments increases for the individual who experiences this situation intensely.

During university years, individuals leave their families and need to gain a place and exist within the social environment they have established. In particular, university students use social media applications extensively to communicate with their social environment, including their classmates (Ophus & Abbitt, 2009). This opportunity of internet and social media applications helps university students to feel that they receive social support from their environments such as distant family and friends (Gemmill & Peterson, 2006).

Studies have demonstrated that FoMO is negatively related to social well-being (Burke et al., 2010), emotional stability, conscientiousness, problematic internet use and well-being (Stead & Bibby, 2017), psychological need satisfaction, and general mood (Przybylski et al., 2013). On the other hand, FoMO is positively related social media engagement (Przybylski et al., 2013), problematic Instagram use (Balta et al., 2020), problematic smartphone use (Elhai et al., 2020, b), pubbing (Balta et al., 2020), anxiety (Balta et al., 2020; Elhai, Gallinari, et al., 2020; Elhai, Yang, et al., 2020; Holte & Ferraro, 2020; Wolniewicz et al., 2020), neuroticism (Balta et al., 2020), depression (Elhai, Gallinari, et al., 2020; Holte & Ferraro, 2020; Wolniewicz et al., 2020), and rumination (Elhai, Yang, et al., 2020).

## Social self-efficacy

Social self-efficacy is defined as an individual’s confidence in the ability to participate in social interactive tasks necessary to initiate and maintain interpersonal relationships in his/her social life (Anderson & Betz, 2001). Social self-efficacy is considered as a necessary skill not only for establishing successful social relationships but also for maintaining mental health (Lin & Betz, 2009). Social self-efficacy was also associated with personality traits. In the research conducted by Mak and Tran (2001), significant positive relationships were found between social self-efficacy and extraversion, openness and conscientiousness. Therefore, the social self-efficacy of individuals who are open to new experiences and extrovert also increases.

Increased social self-efficacy allows the individual to have courage in establishing social relationships. Moreover, individuals who establish positive social relationships gain experience in establishing social relationships. Individuals with high social self-efficacy can use their problem-solving skills effectively in interpersonal relationships (Erözkan, 2013). Therefore, they can also effectively cope with the problems they encounter in electronic social environments. Individuals who have established successful social relationships expect similar results in their subsequent social relationship experiences (Bakioğlu, 2020;Bakioglu & Turkum, 2017 ; Wright & Perrone, 2010).

Studies have demonstrated that social self-efficacy is negatively related academic stress, and interpersonal relationship stress (Chiu, 2014), internet addiction (Bakioğlu, 2020; Gazo et al., 2020; Severino & Craparob, 2013), loneliness (Bakioğlu, 2020; Gazo et al., 2020) pathological gambling (Passanisi et al., 2020), game addiction (Jeong & Kim, 2011), online game addiction (Duman & Ozkara, 2019), accepting external influence, and self-alienating (Satici et al., 2013), depression and shyness (Anderson & Betz, 2001). On the other hand, social self-efficacy is positively correlated perceived social support (Adams et al., 2019; Bakioğlu, 2020; Traş & Arslan, 2013), life satisfaction (Bakioglu & Turkum, 2017; Wright & Perrone, 2010), authentic living (Satici et al., 2013), and communication skills and interpersonal problem solving skills (Erözkan, 2013). A negative relationship was found between self-efficacy and FoMO (Erdoğan & Şanlı, 2019; Lee et al., 2020). In the literature, there are no research findings that directly address the relationship between social self-efficacy, and FoMO. However, it can be stated that as individuals’ social self-efficacy increases, they establish and maintain more satisfying relationships and their FoMO level will decrease.

## Satisfaction with life

Previous researchers considered satisfaction with life as the cognitive component of subjective well-being. Satisfaction with life is an assessment of perceived quality of life (Andrews & Withey, 1976; Diener et al., 1985) and satisfaction from different areas of life (Myers & Diener, 1995). In determining the individuals’ satisfaction with life, the meaning they attribute to life, happiness in their daily life, harmony to achieve their goals, feeling physically healthy, positive individual identity, security, economic, and social relations are considered important (Schmitter, 2003).

An individual feels satisfaction when a wish, purpose, or need is met in his/her life. The individual’s satisfaction enables him/her to feel self-sufficient and to establish and maintain social relationships, to set goals, and to reach certain standards in his life (Dem et al., 2016). Moreover, satisfaction with life is the evaluation of individual on his/her family, friends, social relationships, and the feeling of content with his/her own life (Suldo & Huebner, 2006). Being happy in daily life of the individual, feeling that life is meaningful, seeing himself/herself sufficient to achieve his/her goals, having good physical health, meeting the needs of his/her socio-economic situation, and establishing positive social relationships are determined as effective factors in life satisfaction (Keser, 2005).

Studies have demonstrated that life satisfaction is positively related to self-esteem (Cobos-Sanchiz et al., 2020; Saad, 2020), social self-efficacy (Bakioglu & Turkum, 2017; Ditchman et al., 2017; Jeon, 2016; Jian et al., 2018; Zamani & Shirazi, 2020), perceived social support (Cobos-Sanchiz et al., 2020; Malinauskas, 2010), optimism (Oriol et al., 2020; Türküm, 2005), self-control, and positive affection (Oriol et al., 2020). On the other hand, there are negative correlations between life satisfaction and psychological distress (Cobos-Sanchiz et al., 2020; Satici et al., 2020; Zhang et al., 2020), loneliness (Bakioglu & Turkum, 2017; Mellor et al., 2008), impulsivity, problematic video gaming (Cudo et al., 2020), and negative affectation (Deniz et al., 2012). Studies have shown that as individuals’ social self-efficacy increases (starting and maintaining social relationships, etc.), their life satisfaction also increases (Bakioglu & Turkum, 2017; Ditchman et al., 2017; Jeon, 2016; Jian et al., 2018; Zamani & Shirazi, 2020). Moreover, life satisfaction was found to be associated with FoMO. Studies have found that FoMO is negatively associated with life satisfaction. As the FoMO level increases, life satisfaction decreases (Can & Satici, 2019; Giagkou et al., 2018; Hızarcı, 2018; Przybylski et al., 2013; Sette et al., 2020).

## Aims

University years coincide with the young adulthood period. In these years, individuals take steps to establish, strengthen, and maintain long-term social relationships. When the individual realizes that social relations continue outside his/her control while establishing social relationships, they want to be involved in it. They think they may miss new developments when they are not in the same environment with his/her friends. Therefore, the fear of missing out on developments may cause him/her to try to be more assertive in his/her social relationships and to force his/her friends. All these developments allow the level of FoMO to decrease as the social self-efficacy level of the individual increases. Research results show that there is a negative relationship between social self-efficacy and satisfaction with life, and FoMO. Therefore, as the social efficacy of the individual increases, the FoMO level decreases and satisfaction with life can increase.

In the light of all this information, it can be said that as the social self-efficacy level of university students’ increases, their FoMO level decreases and their satisfaction with life increases. Moreover, a negative relationship is expected between social self-efficacy and life satisfaction, and FoMO. A positive relationship is expected between social self-efficacy and life satisfaction. In this study, it was aimed to examine whether FoMO mediates the relationship between social self-efficacy and satisfaction with life.

## Hypotheses

**Fig. 1:**
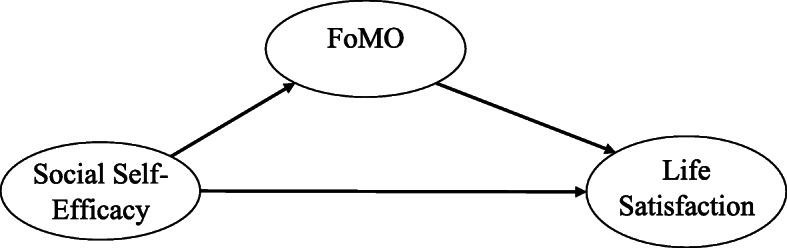
The hypothesized model

Based on the literature presented, we have put forward the following hypotheses. Each of these hypotheses represents a portion of Fig. 1.

H1. Social self-efficacy will be positively related to life satisfaction.

H2. Social self-efficacy will be negatively related to FoMO.

H3. FoMO will be negatively related to life satisfaction.

H4. The relationships between social self-efficacy and life satisfaction will be mediated by FoMO.

## Method

### Participants

The data were collected through online surveys. It took about 20-25 min for participants to fill in the survey. The data were collected from 323 volunteer undergraduate students at a state university in Turkey. Of all the participants, 214 (66.3%) were females and 109 (33.7%) were males whose ages ranged from 18 to 32 years (*M* = 21.52, *SD* = 2.69). The descriptive information of the participants is presented in Table 1.

**Table 1:** Descriptive information of the participants

	Frequency	%
Gender		
Female	214	66.3
Male	109	33.7
Number of social media accounts		
1-2	137	42.4
3-4	137	42.4
5 and more	49	15.2
How often to check Facebook notifications during the day		
Every 0-30 min	134	41.5
Every 31-59 min	12	3.7
Every 1-2 h	9	2.8
More than 2 h	47	14.6
Not checking	40	12.4
Does not have an account	81	25.1
How often to check Twitter notifications during the day		
Every 0-30 min	86	26.6
Every 31-59 min	53	16.4
Every 1-2 h	50	15.5
More than 2 h	56	17.3
Not checking	17	5.3
Does not have an account	61	18.9
How often to check Instagram notifications during the day		
Every 0-30 min	52	16.1
Every 31-59 min	60	18.6
Every 1-2 h	90	27.9
More than 2 h	97	30.0
Does not have an account	24	7.4
How often to check YouTube notifications during the day		
Every 0-30 min	153	47.4
Every 31-59 min	21	6.5
Every 1-2 h	39	12.1
More than 2 h	110	34.1

### Measures

#### Fear of missing out scale

Fear of missing out of the participants was assessed using the Fear of Missing out Scale (FoMOs; Przybylski et al., 2013). Participants assess 10 items (e.g., “I fear my friends have more rewarding experiences than me.”) on a 5-point Likert-type scale ranging from 1 (not at all true of me) to 5 (extremely true of me), with higher scores indicating higher levels of FoMO. In this study, the Turkish version of the FoMO scale was used (Can & Satici, 2019). The Turkish version of the FoMO scale internal consistency coefficient (*α* = .78) and test re-test reliability (.86), was found to be good. Moreover, the construct validity of the scale was found to be excellent (*χ*^*2*^/*df* = 1.79, GFI = .92, CFI = .90, SRMR = .006, and RMSEA = .07; Can & Satici, 2019). In the present study, the Cronbach’s *α* was good (.81). The construct validity of the scale for present study was found to be excellent (*χ*^*2*^/*df* = 2.04, GFI = .96, CFI = .97, SRMR = .04, and RMSEA = .05).

#### Social efficacy and social outcome expectation scale

Social self-efficacy of the participants was assessed using the Social Efficacy and Social Outcome Expectation Scale (SEOES; Wright et al., 2013). Participants assess 19 items (e.g., “I am confident in my skills to be in social relationships” for social efficacy and “Doing nice things for others will increase my social relationships” for social outcome expectation) and two components (social efficacy and social outcome expectation) on a 5-point Likert-type scale ranging from 1 (strongly disagree) to 5 (strongly agree), with higher scores indicating higher levels of social efficacy. In this study, the Turkish version of the SEOES was used (Bakioglu & Turkum, 2017). The Turkish version of the SEOES internal consistency coefficient (*α* = .92 for social efficacy, *α* = .81 for social outcome expectation, and *α* = .91 for total) and test re-test reliability (.90), was found to be excellent. Moreover, the construct validity of the scale was found to be excellent (*χ*^*2*^/*df* = 2.76, GFI = .89, CFI = .98, SRMR = .02, and RMSEA = .07; Bakioglu & Turkum, 2017). In the present study, the Cronbach’s α was very good (*α* = .92 for social efficacy and *α* = .77 for social outcome expectation). The construct validity of the scale for present study was found to be acceptable (*χ*^*2*^/*df* = 3.60, GFI = .86, CFI = .89, SRMR = .05, and RMSEA = .08).

#### Satisfaction with life scale

The life satisfaction of the participants was assessed using the Satisfaction with Life Scale (SWLS; Diener et al., 1985). Participants assess 5 items (e.g., “The conditions of my life are excellent.”) on a 5-point Likert-type scale ranging from 1 (strongly disagree) to 5 (strongly agree), with higher scores indicating higher levels of life satisfaction. In this study, the Turkish version of the SWLS was used (Dağlı & Baysal, 2016). The Turkish version of the SWLS internal consistency coefficient (*α* = .88) and test re-test reliability (.97), was found to be excellent. Moreover, the construct validity of the scale was found to be excellent (*χ*^*2*^/*df* = 1.17, GFI = .99, AGFI = .97, CFI = 1.00, NFI = .99, NNFI = .1.00, SRMR = .019, and RMSEA = .03; Dağlı & Baysal, 2016). In the present study, the Cronbach’s *α* was good (.78). The construct validity of the scale for present study was found to be excellent (*χ*^*2*^/*df* = 1.52, GFI = .99, CFI = .99, SRMR = .02, and RMSEA = .04).

#### Data analysis

We carried out the analyzes in two stages. First, we examined normality, reliability, and the relationships between variables. Second, we tested the structural equation model. We used the maximum likelihood estimation technique in the structural equation model. In addition, we used the parceling technique to reduce the number of observed variables and to increase reliability and normality (Nasser-Abu Alhija & Wisenbaker, 2006). We created two parcel for the FoMO scale (Little et al., 2002). We used various fit indices (e.g., *χ*^2^/*df* < 5, CFI, TLI, GFI, IFI > .90, SRMR and RMSEA < .08, Hu & Bentler, 1999; MacCallum et al., 1996; Tabachnick & Fidell, 2007) to evaluate the structural equation model.

In this study, we used bootstrapping analysis for mediation analysis (Preacher and Hayes, 2008). Bootstrapping analysis allows to test whether direct and indirect effects are significant in bigger samples (MacKinnon et al., 2004). In the bootstrapping analysis, 10,000 resampling and 95% confidence interval (CI) were used. We conducted the analysis of the data by using the SPSS® Statistics 21.00 and IBM SPSS® Amos 23.00 software.

## Results

Table 2 displays bivariate descriptive statistics, Pearson correlation coefficients, and reliabilities for the study variables. Social self-efficacy was found to be positively associated with life satisfaction (*r* = .42 and .34, *p* < .001) (H1) and negatively associated with FoMO (*r* = −.45 and −.37, *p* < .001) (H2). FoMO was found to be negatively associated with life satisfaction (*r* = − .41, *p* < .001) (H3). In short, all the variables of the study were significantly associated with each other.

**Table 2:** Correlation matrix of primary study variables

Variable	1	2	3	4	*α*	*M*	*SD*	Skewness	Kurtosis
1. Social efficacy	-				.92	49.80	6.57	−.52	.31
2. Social outcome	.54^**^	-			.77	24.08	2.47	−.13	.23
3. FoMO	−.45^**^	−.37^**^	-		.81	22.83	6.16	.53	−.13
4. Life satisfaction	.42^**^	.34^**^	−.41^**^	-	.78	15.13	3.27	−.13	−.04

Note. ^**^Correlation is significant at *p* < 0.001 (2-tailed)

After preliminary analysis, we analyzed the normality assumptions. In the findings, we found that the skewness values ranged from −.52 to .53, and the kurtosis values ranged from .13 to .31. We found that the reliability coefficients were above .70 and these values were acceptable. We found that the entire Mahalanobis distance was less than 3, the variance inflation factor values ranged from 1.28 to 1.57, the tolerance values ranged from .63 to .69, and the Durbin Watson value was 1.64. All of these results showed that there was no multicollinearity and residual problem and that all of Field’s (2016) assumptions were met.

**Fig. 2:**
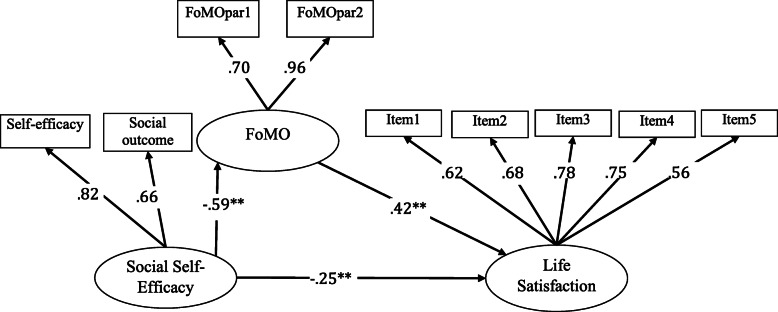
Mediation for social self-efficacy on life satisfaction via FoMO

We examined all the path coefficients in the model and found that it was significant. Social self-efficacy predicted life satisfaction positively (*β* = .42, *p* <.001) (supporting H1) and FoMO negatively (*β* = −.59, *p* <.001) (supporting H2). In addition, FoMO predicted life satisfaction negatively (*β* = −.25, *p* <.001) (supporting H3). Among the mediation hypotheses, FoMO mediated the relationship between social self-efficacy and life satisfaction, *β =* −.25, *p* < .001 (supporting H4) (Fig. 2).

We examined all fit indices in the structural equation model of the research and found that all of them indicated perfect fit. (*χ*^2^_(22, *N* = 323)_ = 39.83, *p* <.001; *χ*^2^/*df* = 1.81; GFI = .97; CFI = .98; NFI = .96; TLI = .97; SRMR = .031; RMSEA =.05). All these results show that the hypothetical structural equation model has been validated.

In this study, we tested the mediating role of FoMO in the relationship between social self-efficacy and life satisfaction through bootstrapping procedure. In the bootstrap procedure (coefficients and confidence intervals for direct and indirect effects), we used 10,000 resampling and a 95% confidence interval (CI). The results obtained are presented in Table 3.

**Table 3:** Indirect effect of social self-efficacy on life satisfaction via FoMO

Paths	Coefficient	95% C. I.	
		Low limit	Up limit
**Direct effect**			
Social self-efficacy ➔ Life satisfaction	.42	.61	.24
Social self-efficacy ➔ FoMO	−.59	−.70	−.48
FoMO ➔ Life satisfaction	−.25	−.41	−.08
**Indirect effect**			
Social self-efficacy ➔ FoMO ➔ Life satisfaction	−.25	−.40	−.13

When Table 3 was examined, we saw that all direct effects were significant. We also saw that the indirect effects in the hypothetical model of the research were confirmed [effect = −.25; CI = (−.40, −.13)]. According to the results of the bootstrapping analysis, we can say that undergraduate students’ social self-efficacy predicts life satisfaction through FoMO.

## Discussion

In this study, the mediating role of FoMO in the relationship between social self-efficacy and satisfaction with life was examined. For this purpose, answers to the hypotheses determined in the study were sought. All path coefficients in the structural equation model of the study were found to be significant (Hu & Bentler, 1999). Discussion of the research hypotheses is presented below, respectively.

Firstly, a positive relationship was found between social self-efficacy and satisfaction with life (supporting H1). Examining the literature, it was seen that studies were supporting a positive relationship between social self-efficacy and satisfaction with life (Bakioglu & Turkum, 2017; Ditchman et al., 2017;Jeon, 2016 ; Jian et al., 2018 ; Zamani & Shirazi, 2020). The increase in social self-efficacy of individuals in starting and maintaining social relations also contributes to the development of social relations. It allows individuals with developed social relations to get more satisfaction from their lives and to be happy (Jeon, 2016; Jian et al., 2018; Zamani & Shirazi, 2020). This finding indicates that as individuals’ social self-efficacy increase, their satisfaction with life also increases.

Secondly, a negatively significant relationship was found between social self-efficacy and FoMO (supporting H2). When this finding of the study is evaluated, no research was found in the literature that addresses the relationship between social self-efficacy and FoMO. However, studies examining the relationship between self-efficacy and FoMO have shown a negative relationship between these two variables (Erdoğan & Şanlı, 2019; Lee et al., 2020). Moreover, it is thought that as the social self-efficacy of the individual increases, they may not follow social relationships that they are not involved in. Therefore, the increase in the social self-efficacy of the individual may allow the FoMO level to decrease. As the FoMO level of individuals increases, they spend more time on the Internet and follow what is happening in the virtual environment. When individuals see that they are not included in the activities organized by their acquaintances in virtual environments, they may avoid social relations. Thus, the social self-efficacy of individuals to initiate and maintain social relations may also decrease.

Third of all, a significant negative correlation was found between FoMO and satisfaction with life (supporting H3). Many studies in the related literature show a negative relationship between FoMO and satisfaction with life (Can & Satici, 2019; Giagkou et al., 2018; Hızarcı, 2018; Przybylski et al., 2013; Sette et al., 2020). This finding indicates that as the individual’s FoMO level increases, his/her life satisfaction decreases.

Finally, it was discovered that FoMO has a mediating role in the relationship between social self-efficacy and satisfaction with life (supporting H4). When this finding is analyzed, it is seen that the bilateral relationships (H1, H2, H3) between the variables of the study give significant results. On the other hand, it has been observed that there is no structural equation research directly addressing the relationships between social self-efficacy, satisfaction with life and FoMO, which are discussed in this study. Evaluating the structural equation model of the study, the increase in the social self-efficacy of the individual, which refers to establishment and maintenance of new social relationships, decreases the FoMO, which expresses the anxiety about missing developments (Erdoğan & Şanlı, 2019; Lee et al., 2020) and consequently increases the satisfaction with life (Bakioglu & Turkum, 2017; Ditchman et al., 2017; Jeon, 2016; Jian et al., 2018; Zamani & Shirazi, 2020). University years are the times when individuals stay on their own away from their family, but maintain their social relations with their family and friends both in person and in virtual environments (Gemmill & Peterson, 2006; Ophus & Abbitt, 2009). The individual becomes less dependent on virtual environments when they meet their basic need for social relationships. Therefore, his/her FoMo level decreases and satisfaction with life increases.

This study investigating the mediating role of FoMo levels in the relationship between university students’ social self-efficacy and life satisfaction has some limitations. First of all, this research is a cross-sectional and quantitative research conducted in the structural equation model. It is recommended to conduct research in longitudinal and experimental designs and supported by qualitative research data. Secondly, this research was carried out by a group of university students in Turkey. Therefore, studies can be conducted with larger samples with different cultural characteristics. Another limitation of this study is that 66% of the study group consisted of women. In future studies, the mean scores of male and female participants can be compared.

Domain experts and practitioners can prepare and implement programs that can increase the social self-efficacy skills of individuals, especially at university ages, in cooperation with universities and non-governmental organizations. Mental health professionals and domain experts can provide protection to individuals from the negative effects of FoMO by preparing psycho-education programs in a preventive context and increasing their skills in initiating and maintaining relationships.

## Conclusion

In this study, it was found that FoMO has a mediating role in the relationship between social self-efficacy and satisfaction with life. Increasing the social self-efficacy of the individual allows him/her to establish and maintain new relationships. Moreover, individuals who establish successful social relationships may prefer to meet this need less in virtual environments. Thus, the door of the individual to be more satisfied with life will be opened by establishing real social relationships. During the times when university students are away from their families, thanks to the real relationships they establish, they can be prepared for life better and contribute to their personal and professional development.

## Data Availability

There is no availability of data and materials.

## Abbreviations

FoMOs: Fear of Missing out Scale
SEOES: The Social Efficacy and Social Outcome Expectation Scale
SWLS: Satisfaction with Life Scale
β: Standardized coefficient
